# An Unusual Case of Severe Hyperglycemia in an 82-Year-Old Man During Acute COVID-19 Infection

**DOI:** 10.7759/cureus.108655

**Published:** 2026-05-11

**Authors:** Cooper Bullough, Leslie Matherne, Jared Rhinehardt

**Affiliations:** 1 Internal Medicine, Quillen College of Medicine, Johnson City, USA; 2 Internal Medicine, East Tennessee State University Quillen College of Medicine, Johnson City, USA; 3 Family Medicine, East Tennessee State University Quillen College of Medicine, Johnson City, USA

**Keywords:** ace-2 receptor, new onset diabetes after covid, post covid-19 complication, post covid-19 diabetes, tmprss2

## Abstract

Severe hyperglycemia during acute COVID-19 infection is an uncommon but clinically significant presentation. Here, we present an unusual case of an 82-year-old COVID-19-positive man who presented with severe hyperglycemia following several days of COVID-19 symptoms, with an admission HbA1c of 14.7%. He received standard treatment for a working diagnosis of diabetic ketoacidosis (DKA), improved clinically, and was discharged after five days, but continued to require exogenous insulin to maintain appropriate fasting and postprandial blood glucose levels. This case represents possible COVID-19-associated metabolic decompensation in previously unrecognized type 2 diabetes. It highlights the importance of monitoring patients with COVID-19 for severe hyperglycemia and metabolic decompensation, particularly in elderly individuals, those with metabolic risk factors, or those with severe clinical presentations.

## Introduction

Post-COVID diabetes has emerged as an important yet underrecognized sequela of COVID-19 [1]. There is an increased risk of diabetes after COVID-19 infection compared with before infection [2]. One meta-analysis, which included nine studies and accounted for over 40 million patients, showed an estimated relative risk (RR) of diabetes of 1.62 (95% confidence interval (CI): 1.45-1.80) [3]. The mechanisms appear to be multifactorial and are poorly understood [1,2]. The clinical manifestation of post-COVID diabetes also varies, with some cases resembling type 1 diabetes (T1D) and others resembling type 2 diabetes (T2D), with the T2D manifestation appearing to be more common [4,5]. The overall incidence of new-onset diabetes after COVID-19 infection is low. According to one study, it occurs in approximately 1.4% of cases [5]. However, given the large number of COVID-19 infections, this could translate to a substantial absolute number of diabetes cases, making post-COVID diabetes a meaningful public health issue that remains underrecognized. Post-COVID diabetes cases are also unique in that it is difficult to determine whether COVID-19 itself drives the underlying pathophysiology, unmasks or exacerbates preexisting metabolic dysfunction, or represents a combination of these processes. This underscores the need for additional research to more clearly determine causality. The following case report highlights one such occurrence of severe hyperglycemia during acute COVID-19 infection.

## Case presentation

This case highlights an 82-year-old man who gave verbal consent for this case report. He presented to an outpatient family medicine clinic with several days of chest pain and feeling generally unwell. Medical history included hypertension, hyperlipidemia, non-alcoholic steatosis of the liver, and a stable chronic deep vein thrombosis of the lower extremity managed with chronic anticoagulation. He denied tobacco or alcohol use. The patient had never been diagnosed with diabetes in the past. There was a family history of type 2 diabetes in the patient’s mother and sister. The patient’s medications included amlodipine 2.5 mg daily, atorvastatin 40 mg daily, lisinopril 20 mg daily, and warfarin 5 mg daily. The patient had not recently received corticosteroids.

In the clinic, the patient reported chest pain, generalized weakness, shortness of breath, and nausea for several days prior. Blood pressure was 97/69 mm Hg, and oxygen saturation was 89% on room air. The patient was then referred to the emergency department (ED).

In the ED, the patient was afebrile, normotensive, and not in respiratory distress. His body mass index (BMI) was 26.07. The patient was alert and oriented to person, place, and time and had no focal neurologic deficits. The patient appeared well-nourished and had moist mucous membranes. Capillary refill was less than 2 seconds. The initial complete metabolic panel (CMP), total bilirubin, beta-hydroxybutyrate, and pertinent complete blood count (CBC) results are shown in Table 1.

**Table 1:** Initial laboratory workup BUN: blood urea nitrogen, eGFR: estimated glomerular filtration rate, ALT: alanine aminotransferase, AST: aspartate aminotransferase

Laboratory parameter	Result	Reference range
Sodium	118 mmol/L	135-145 mmol/L
Sodium corrected for hyperglycemia	130 mmol/L	135-145 mmol/L
Potassium	5.4 mmol/L	3.5-5.1 mmol/L
Chloride	83 mmol/L	98-107 mmol/L
CO2	21 mmol/L	22-30 mmol/L
Glucose	880 mg/dL	70-99 mg/dL
BUN	56 mg/dL	9-20 mg/dL
Creatinine	1.61 mg/dL	0.66-1.25 mg/dL
BUN:creatinine	34.8	7.3-21.7
Calcium	11.1 mg/dL	8.6-10.3 mg/dL
Anion gap	14 mmol/L	7-15 mmol/L
eGFR	42 ml/min/SA	≥60 ml/min/SA
Total protein	7.6 g/dL	6.3-8.2 g/dL
Albumin	4.8 g/dL	3.5-5.0 g/dL
ALT	64 U/L	18-62 U/L
AST	58 U/L	18-39 U/L
Calculated osmolality	297 mOsm/L	275-295 mOsm/L
Alkaline phosphatase	97 U/L	38-126 U/L
Bilirubin, total	2.1 mg/dL	0.2-1.3 mg/dL
Beta-hydroxybutyrate	2.37 mmol/L	0.2-0.27 mmol/L
White blood cells	11.8 K/µL	3.5-11.0 K/µL
Hematocrit	39.70%	41.0%-51.0%
Hemoglobin	13.7 g/dL	13.9-16.8 g/dL

Serial troponins were negative. Chest X-ray and electrocardiogram were both unremarkable. The severe acute respiratory syndrome coronavirus 2 (SARS-CoV-2) swab was positive. Urinalysis, urine ketones, and arterial blood gases were not obtained.

The patient’s baseline creatinine was approximately 0.9 mg/dL. Given the patient’s creatinine of 1.61 and increased BUN:creatinine ratio, pre-renal azotemia was likely. Hemoglobin A1c was 14.7%. Serum glucagon and C-peptide were ordered. The patient was started on the Glucommander protocol for a working diagnosis of diabetic ketoacidosis (DKA), fluid resuscitation was initiated, and he was admitted. About 12 hours into admission, his glucose had improved to 101 mg/dL, his creatinine had begun to downtrend from 1.61 mg/dL to 1.52 mg/dL, and his CO2 had increased from 21 mmol/L to 26 mmol/L, all suggestive of improvement of metabolic function, renal function, and acid-base status.

During admission, he received a five-day course of remdesivir for the treatment of COVID-19. A computed tomography (CT) scan of the abdomen and pelvis was negative for pancreatic masses or evidence of pancreatitis. A lipase level was slightly elevated at 313 U/L (normal range: 23-300 U/L). Autoimmune diabetes markers were not obtained. He clinically improved throughout his hospitalization. However, the patient continued to require exogenous insulin to maintain appropriate fasting and postprandial blood glucose levels. He was discharged after five days of treatment and received diabetes education and counseling on medication usage and diet. The plan at the time of discharge was 20 units of insulin glargine once daily and 8 units of insulin lispro three times per day with meals and close outpatient follow-up for further evaluation. Shortly after discharge, serum glucagon was found to be elevated at 279 pg/mL (normal range: 70-160 pg/mL), and C-peptide was found to be normal at 2.3 ng/mL (normal range: 1.1-2.6 ng/mL).

In the months following discharge, the patient was adherent to close follow-up and to his new medication regimen. Metformin 500 mg twice daily was added for further glucose control. Approximately three months after discharge, the patient’s A1c had decreased to 6%. His insulin lispro was decreased to five units twice daily, and insulin glargine and metformin were continued at the previously stated doses. From a diabetic perspective, the patient is currently doing well on this regimen without further need for admission related to hyperglycemia.

## Discussion

This was an unusual case of severe hyperglycemia during COVID-19 infection in an 82-year-old man. This case was also unique in that the patient had a clinical presentation that did not appear to perfectly align with DKA or hyperosmolar hyperglycemic state (HHS). Table 2 compares the patient’s parameters to established criteria [6] for the diagnoses of DKA and HHS.

**Table 2:** Comparison of case patient to established diagnostic criteria of DKA and HHS DKA: diabetic ketoacidosis, HHS: hyperosmolar hyperglycemic state

Parameter	Case patient	DKA	HHS
Glucose	880 mg/dL	≥200 mg/dL	≥600 mg/dL
Beta-hydroxybutyrate	2.37 mmol/L	≥3.0 mmol/L	<3.0 mmol/L
Urine ketones	Unavailable	2+ or greater	less than 2+
pH	Unavailable	<7.3	≥7.3
Bicarbonate	~21 mmol/L (based on CO2)	<18 mmol/L	≥15 mmol/L
Anion gap	14 mmol/L	>10-12 mmol/L	Variable
Osmolality	297 mOsm/L	Variable	>300 mOsm/L
Mental status	Alert	Usually alert	Usually altered
Onset	Days	Hours to days	Days to a week

The patient’s extremely high glucose of 880 mg/dL, beta-hydroxybutyrate of 2.37 mmol/L, and bicarbonate of approximately 21 mmol/L are suggestive of HHS; however, the patient lacked the osmolality greater than 300 mOsm/L and the mental status changes that would be expected with HHS. The patient’s anion gap met criteria for DKA; however, the unavailability of urine ketones and pH makes it impossible to assign the patient a diagnosis of DKA. A mixed picture of DKA and HHS occurs in approximately one-third of hyperglycemic emergencies [6]. Given that the patient does not fully meet criteria for either diagnosis, this case could represent one such case of mixed DKA and HHS.

Another unique aspect of this case is that the patient did not have a formal diagnosis of diabetes prior to his COVID-19 infection. He had been followed in clinic for approximately 12 years, and his highest blood glucose reading on routine complete metabolic panels (CMPs) over that time had been 111 mg/dL. He never had consecutive CMPs showing a glucose greater than 100 mg/dL, and it is unknown if he was fasted for these CMPs. He had not previously reported symptoms of chronic hyperglycemia, such as neuropathic symptoms, vision changes, polyuria, polydipsia, or changes in weight. A1c was not followed over the 12 years the patient was in our clinic. He arrived without a diagnosis of diabetes and was already 70 years old; therefore, the decision was jointly made between the patient and physician to defer screening. Therefore, baseline A1c data were unavailable. The patient’s A1c was 14.7% upon arrival. While this normally represents a three-month average, it has been previously described that this number can be significantly distorted due to several days of severe hyperglycemia prior to admission, causing disproportionate amounts of glycation [7], leading to a falsely high reading. However, it is unclear if an increase of such a large magnitude could be caused by short-term hyperglycemia. The patient did have mild hepatic steatosis seen incidentally on a right upper quadrant ultrasound six years prior to the admission discussed herein, but the report of the CT obtained during this admission did not comment on hepatic steatosis. No other prior imaging was obtained on the patient. Cumulatively, the lack of a baseline A1c, possible hepatic steatosis seen on ultrasound, a significantly elevated A1c at time of admission, and the unavailability of autoimmune diabetes markers made it difficult to determine if this was a case of prior undiagnosed T2D unmasked by a response to COVID-19 infection or if this was a case of new-onset diabetes following COVID-19.

Post-COVID diabetes can manifest in a pattern similar to T1D or T2D. Given the patient’s normal C-peptide levels, he seems to have maintained his ability to produce endogenous insulin, which appears more consistent with a T2D pattern [8]. However, this result should be interpreted with some caution, as acute illness can increase C-peptide levels. A repeat C-peptide level was not obtained after resolution of the acute illness, making it difficult to determine the amount of endogenous insulin production that the patient retained. Furthermore, the patient’s BHB was only 2.37 mmol/L, which is also more consistent with a T2D pattern; levels over 3 mmol/L are generally expected with new-onset T1D [9]. The long-term management of the patient’s dysglycemia will be similar to non-COVID-associated T2D, with consistent A1c monitoring and medication adjustments as needed.

**Figure 1:**
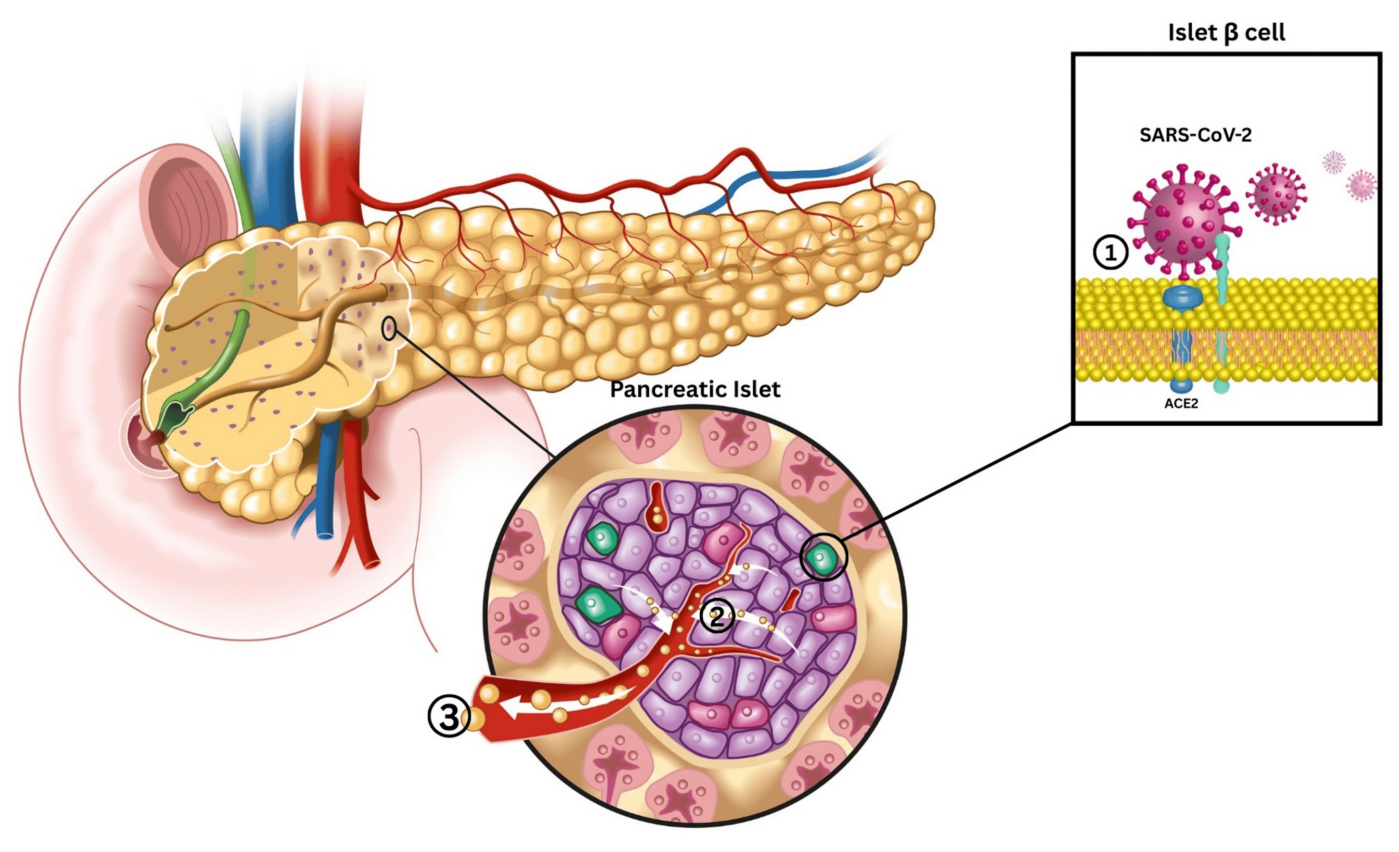
Proposed mechanisms via which COVID-19 induces pancreatic dysfunction and diabetes 1: SARS-CoV-2 seems to be able to induce direct cytotoxicity and cellular dysfunction via the ACE2 and TMPRSS2 receptors. 2: Local inflammation due to cytokine response to the virus also appears to play a role in mediating the damage to pancreatic cells. 3: Cytokine response associated with COVID-19 leads to systemic inflammation, which can drive hyperglycemia and insulin resistance. ACE2: angiotensin-converting enzyme 2, TMPRSS2: transmembrane protease serine 2 The figure included in this article is an original image created by the authors using Adobe and Canva software. Generative AI was not used in the creation or enhancement of this figure.

There are multiple mechanisms through which SARS-CoV-2 is believed to induce T2D. SARS-CoV-2 seems to be able to directly infect pancreatic beta-cells, specifically via the angiotensin-converting enzyme 2 (ACE2) and transmembrane protease serine 2 (TMPRSS2) receptors, inducing direct cytotoxicity and cellular dysfunction (see label 1 in Figure 1) [10]. A local inflammatory cascade caused by cytokine response to the virus also appears to play a role in mediating the damage to pancreatic cells (see label 2 in Figure 1) [11]. Furthermore, the exaggerated cytokine response associated with COVID-19 is known to induce systemic inflammation, leading to increased insulin resistance, disrupting metabolic regulation and causing hyperglycemia (see label 3 in Figure 1) [10]. The complex interplay of these responses to SARS-CoV-2 is what likely drives the induction of diabetes after infection; however, more longitudinal research is needed to fully understand the post-acute sequelae of COVID-19.

## Conclusions

This case highlights an unusual presentation of severe hyperglycemia during acute COVID-19, which required insulin therapy at discharge. Clinicians should remain vigilant for metabolic complications such as DKA, HHS, or a mix thereof in patients with acute COVID-19, as early recognition and treatment are critical. This is especially true for elderly patients or patients with other metabolic risk factors, and is also not limited to patients with a prior diagnosis of diabetes. Further research is needed to better understand the mechanisms and long-term implications of COVID-19-associated metabolic decompensation.
